# Molecular Cloning and Characterization of a Xanthone Prenyltransferase from *Hypericum calycinum* Cell Cultures

**DOI:** 10.3390/molecules200915616

**Published:** 2015-08-27

**Authors:** Tobias Fiesel, Mariam Gaid, Andreas Müller, Joana Bartels, Islam El-Awaad, Till Beuerle, Ludger Ernst, Sönke Behrends, Ludger Beerhues

**Affiliations:** 1 Institute of Pharmaceutical Biology, Technische Universität Braunschweig, Mendelssohnstraße 1, Braunschweig 38106, Germany; E-Mails: t.fiesel@tu-bs.de (T.F.); m.gaid@tu-bs.de (M.G.); der.andreas.mueller@gmx.net (A.M.); i.el-awaad@tu-bs.de (I.E.-A.); t.beuerle@tu-bs.de (T.B.); 2 Center of Pharmaceutical Engineering (PVZ), Technische Universität Braunschweig, Franz-Liszt-Straße 35 A, Braunschweig 38106, Germany; E-Mails: jo.bartels@tu-bs.de (J.B.); s.behrends@tu-bs.de (S.B.); 3 Institute of Pharmacology, Toxicology and Clinical Pharmacy, Technische Universität Braunschweig, Mendelssohnstraße 1, Braunschweig 38106, Germany; 4 Central NMR Laboratory, Technische Universität Braunschweig, Hagenring 30, Braunschweig 38106, Germany; E-Mail: l.ernst@tu-bs.de

**Keywords:** aromatic prenyltransferase, xanthone, *Hypericum*, membrane-bound enzyme, Alzheimer’s disease

## Abstract

In plants, prenylation of metabolites is widely distributed to generate compounds with efficient defense potential and distinct pharmacological activities profitable to human health. Prenylated compounds are formed by members of the prenyltransferase (PT) superfamily, which catalyze the addition of prenyl moieties to a variety of acceptor molecules. Cell cultures of *Hypericum calycinum* respond to elicitor treatment with the accumulation of the prenylated xanthone hyperxanthone E. A cDNA encoding a membrane-bound PT (HcPT) was isolated from a subtracted cDNA library and transcript preparations of *H. calycinum*. An increase in the HcPT transcript level preceded hyperxanthone E accumulation in cell cultures of *H. calycinum* treated with elicitor. The HcPT cDNA was functionally characterized by expression in baculovirus-infected insect cells. The recombinant enzyme catalyzed biosynthesis of 1,3,6,7-tetrahydroxy-8-prenylxanthone through regiospecific C–8 prenylation of 1,3,6,7-tetrahydroxyxanthone, indicating its involvement in hyperxanthone E formation. The enzymatic product shared significant structural features with the previously reported cholinesterase inhibitor γ-mangostin. Thus, our findings may offer a chance for semisynthesis of new active agents to be involved in the treatment of Alzheimer’s disease.

## 1. Introduction

Xanthones are a class of specialized (secondary) plant metabolites which serve as defense compounds against pathogenic microorganisms and herbivores. They are either constitutively formed or accumulated in response to infection and provide the producing plant with a chemical barrier [1,2]. Many compounds exhibit appealing pharmacological activities, thereby attracting wide interest [3,4]. The xanthone scaffold commonly undergoes various biosynthetic modifications, such as glycosylation and prenylation. While glycosylated xanthones are widely distributed in the plant family Gentianaceae, prenylated xanthones are typical of Clusiaceae and Hypericaceae [5]. The latter families not only contain monoprenylated compounds, but also form a number of polyprenylated polycyclic metabolites possessing bridged skeletons. A number of these chemically complex molecules exhibit intriguing biological activities [6,7]. As observed for many phenolic constituents, the addition of prenyl residues significantly affects their pharmacological properties [8].

Prenylation of aromatic scaffolds is catalyzed by aromatic prenyltransferase (PT) enzymes, which link the shikimate and/or polyketide pathways in plants with the isoprenoid routes [8]. The enzymes that have been studied at the gene level are integral membrane proteins possessing a number of transmembrane domains [9]. Their detailed understanding may pave the way for their involvement in engineering strategies.

**Figure 1:**
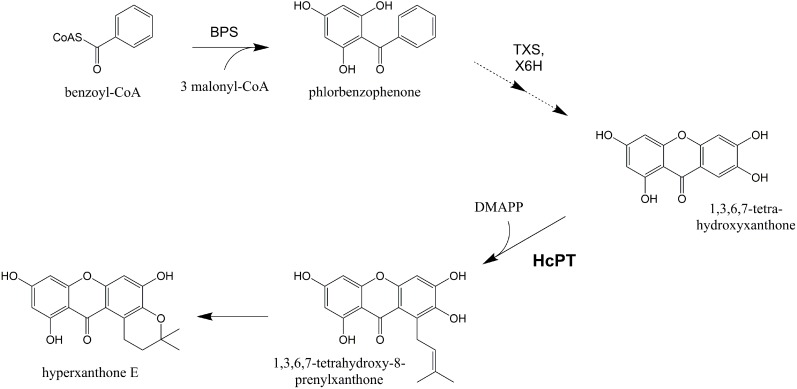
Hyperxanthone E biosynthesis in elicitor-treated *H. calycinum* cell cultures. BPS: benzophenone synthase, TXS: trihydroxyxanthone synthase, X6H: xanthone 6-hydroxylase, DMAPP: dimethylallyl diphosphate, HcPT: *H. calycinum* prenyltransferase.

To gain insight into the biosynthesis of prenylated xanthones, we use elicitor-treated *Hypericum calycinum* cell cultures, which accumulate a monoprenylated xanthone phytoalexin, hyperxanthone E (Figure 1) [10]. *H. calycinum* is closely related to the medicinal plant *Hypericum perforatum* (St. John’s wort; Hypericaceae), extracts of which are widely used for the treatment of mild to moderate depression [11]. The xanthone scaffold is of mixed biosynthetic origin, the two aromatic rings originating from the shikimate and the polyketide pathways (Figure 1). Benzophenone synthase condenses benzoyl-CoA, derived from l-phenylalanine via cinnamoyl-CoA [10], with three molecules of malonyl-CoA [12]. The resulting 2,4,6-trihydroxybenzophenone undergoes cytochrome P450-catalyzed 3′-hydroxylation and heterocyclic ring closure to yield 1,3,7-trihydroxyxanthone [13,14]. Downstream reactions are hydroxylations and prenylations [15,16]. In hyperxanthone E formation, 6-hydroxylation [17] and 8-prenylation are followed by pyran ring formation.

Here we report molecular cloning and functional analysis of a PT cDNA from elicitor-treated *H. calycinum* cell cultures and demonstrate the involvement of the encoded membrane-bound enzyme in the penultimate step of hyperxanthone E biosynthesis.

## 2. Results and Discussion

### 2.1. Isolation and Structural Analysis of a cDNA Encoding an Aromatic Prenyltransferase

**Figure 2:**
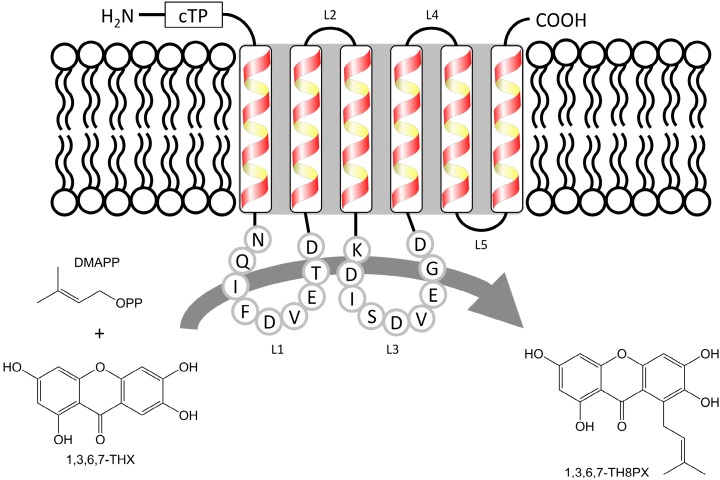
HcPT reaction and predicted topology of the transmembrane domains. The two conserved aspartate-rich motifs, which are characteristic for aromatic prenyltransferases and presumably important for the prenylation reaction [20], are located in the non-membrane loop regions L1 and L3. cTP: putative chloroplast transit peptide, L: loop, DMAPP: dimethylallyl diphosphate, 1,3,6,7-THX: 1,3,6,7-tetrahydroxyxanthone, 1,3,6,7-TH8PX: 1,3,6,7-tetrahydroxy-8-prenylxanthone.

A previously constructed subtracted cDNA library [10] was analyzed for putative aromatic prenyltransferase sequences via the Basic Local Alignment Search Tool (tblastx) of the National Center for Biotechnology Information (NCBI) server [18]. Eleven expressed sequence tags (ESTs) were identified and aligned against aromatic prenyltransferase sequences related to secondary metabolism [19,20,21,22,23]. Six of the identified ESTs shared homology with a single sequence contig. This 464-bp middle fragment encoded a peptide with a characteristic motif of aromatic prenyltransferases (Figure 2). A pool of RNA was isolated from elicitor-treated *H. calycinum* cell cultures, reverse-transcribed, and used as a template for re-amplifying the core fragment with the primer pair 1 + 2 (Table 1, Supplementary Figure S1). Gene-specific forward and reverse primers (3 + 4) then served for 5′ and 3′ rapid amplification of cDNA ends (RACE), which led to cloning of a 1535-bp full-length cDNA. The 1191-bp coding sequence (CDS) was flanked by a 65-bp 5′ untranslated region (UTR) and a 251-bp 3′ UTR plus 28-bp poly(A) tail. The CDS encoded an aromatic prenyltransferase, which was named HcPT and consisted of 396 amino acids with a predicted molecular mass of 43.5 kDa and a pI of 9.7 [24]. HcPT shared highest similarity (38%) with predicted homogentisate solanesyl transferase from *Populus euphratica* (accession number: XP_011047106). The characteristic motifs among aromatic prenyltransferases (motif 1, NQ(I/L)xDxxxD; motif 2, KD(I/L)xDxxGD) were also conserved in HcPT. The amino acid sequence of HcPT contained six putative transmembrane domains, as predicted by the online tool “SOSUI” (Figure 2) [25]. Furthermore, a putative chloroplast transit peptide of 54 amino acids at the *N*-terminus was predicted by the online tools TargetP (score value: 0.837) and ChloroP (score value: 0.563) [26,27,28]. A high probability for targeting HcPT to plastids in plant cells was confirmed by the iPSORT online tool [29]. The same held true for the aromatic prenyltransferases isolated from *Glycine max* [21], *Humulus lupulus* [19,30], *Petroselinum crispum* [9], and *Citrus limon* [31].

**Table 1:** Primer sequences.

No.	Sequence *
*HcPT-specific cloning primers*	
1	5′-TAGTACAAGTGATAAGAAATTCGG-3′
2	5′-TGTACTTAATGGTAAATCGGGTTTG-3′
3	5′-ACTGTATTGACAAGAAGGTTGATGG-3′
4	5′-AACGGCATGTATCTTGATGAACATAGG-3′
*HcCNL-specific primers for transcript level inspection*	
5	5′-TCAGCAGCGTGGAGGTCGAGTCG-3′
6	5′-TTAAAGACGAGACATGGCAAG-3′
*Hc18S-rRNA-specific primers for transcript level normalization*	
7	5′-TGATGGTATCTACTACTCGG-3′
8	5′-AATATACGCTATTGGAGCTGG-3′
*HcPT-specific overexpression primers*	
9	5′-AATGCTAGCATGGAGGTTTCTCGATTGCCATCG-3′
10	5′-AATAAGCTTCTAGATGAAGGGGAGCAAGATAAATTGC-3′
11	5′-ATTGAATTCATGGAGGTTTCTCGATTGC-3′
12	5′-ATTTTAATTAACTAGATGAAGGGGAGCAAGATAAATTGC-3′
13	5′-ATTGAATTCATGGAGGTTTCTCGATTGCCATCG-3′

* Non-natural endonuclease restriction sites are indicated by underlining.

### 2.2. Elicitor-Induced Changes at the Metabolite and Transcript Levels in H. calycinum Cell Cultures

**Figure 3:**
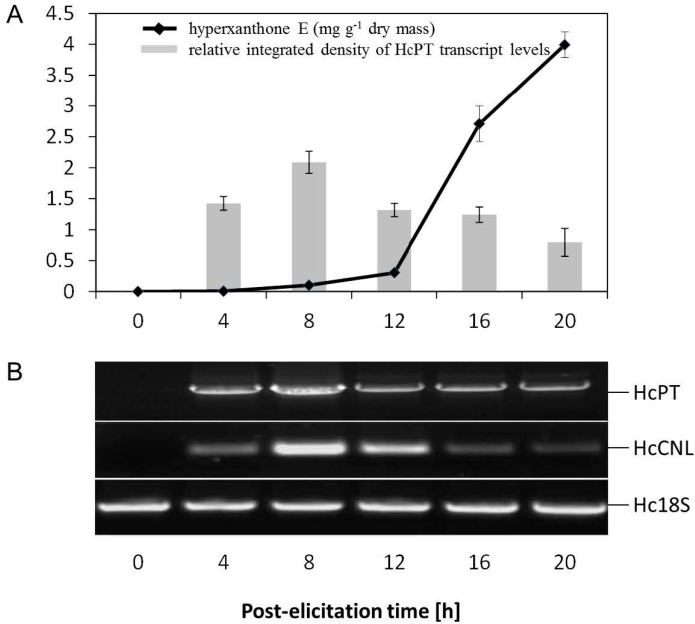
Correlation between gene expression and product formation. (**A**) Changes in hyperxanthone E content and HcPT transcript level in *H. calycinum* cell cultures after the addition of elicitor. Values are means of three biological repeats ± SD; (**B**) Semi-quantitative RT-PCR analysis of HcPT and HcCNL gene expression. Hc18S rRNA transcript levels served for normalization. HcCNL: *H. calycinum* cinnamate:CoA ligase, Hc18S: *H. calycinum* 18S rRNA-derived control.

Yeast extract-treated *H. calycinum* cell cultures were previously shown to form hyperxanthone E, which started to accumulate 12 h after the onset of elicitation and reached the peak level equivalent to 4 mg·g^−1^ dry weight after 20 h [10]. Biosynthesis of hyperxanthone E was preceded by a transient increase in the HcPT transcript level (Figure 3A). A similar expression profile was observed for HcCNL, whose gene product directs the carbon flow to benzenoid/xanthonoid metabolism (Figure 3B). Changes in the transcript levels were studied by semi-quantitative reverse transcription (RT)-PCR, the conditions being optimized as described previously [10]. The sizes of the PCR products upon use of HcPT and HcCNL gene-specific primers (1 + 2 and 5 + 6, respectively; Table 1, Supplementary Figure S1) were 464 and 389 bp, respectively. Following elicitation, both transcripts were detectable after four hours and peaked at eight hours. To ensure equal template amounts, *H. calycinum* 18S rRNA (amplified with the primers 7 + 8) served as control. The absence of detection of HcPT and HcCNL transcripts before elicitation (0 h) as well as their increase after the onset of elicitation provide evidence for the involvement of these enzymes in hyperxanthone E biosynthesis.

### 2.3. Functional Expression of HcPT

HcPT was predicted to contain multiple transmembrane α-helices, suggesting a membranous and hydrophobic protein character. Establishment of this type of protein in prokaryotes is a challenge, contrary to the β-barrel proteins [32], with rare exceptions being reported [33,34]. The presence of α-helical membrane-spanning domains requires a special, *i.e*., eukaryotic, expression system to yield correctly folded protein and circumvent cell death. However, handling of such a hydrophobic protein is a challenge, even in a eukaryotic expression system. Microsomes from yeast (*Saccharomyces cerevisiae*) cells expressing the HcPT coding sequence failed to prenylate the target substrate. Similar observations were made with aromatic prenyltransferases from *Petroselinum crispum* and glycinol-4-dimethylallyltransferase from *Glycine max* [9,21]. An alternative strategy is to produce HcPT in a baculovirus insect cell (*Spodoptera frugiperda*, *Sf9*) expression system. *p-*Hydroxybenzoate:geranyltransferase—a membrane-bound enzyme involved in shikonin biosynthesis in *Lithospermum erythrorhizon—*exhibited ~1000-fold increased activity upon production in the baculovirus insect cell system compared to expression in yeast [22].

**Figure 4:**
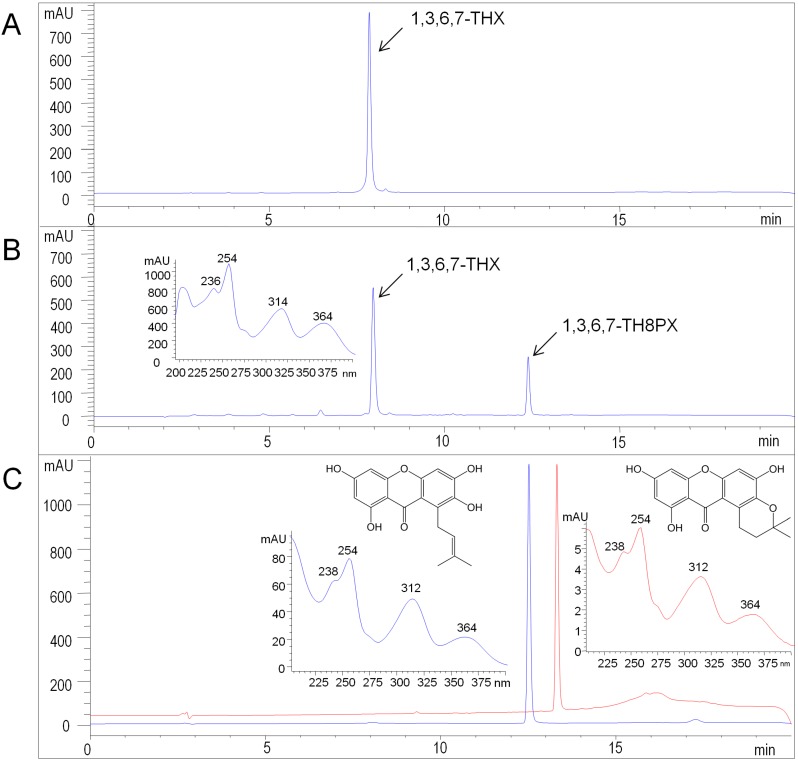
HPLC-DAD analysis of HcPT assays. (**A**) Incubation with denatured protein; (**B**) Standard assay incubated for 30 min. Insert: UV spectrum of the enzymatic product 1,3,6,7-TH8PX; (**C**) Preparative enzymatic synthesis of 1,3,6,7-TH8PX (**blue**) and chromatography of authentic hyperxanthone E (**red**). Inserts: UV spectra. 1,3,6,7-THX: 1,3,6,7-tetrahydroxyxanthone, 1,3,6,7-TH8PX: 1,3,6,7-tetrahydroxy-8-prenylxanthone. Detection wavelength: 254 nm.

The microsomal fraction from insect cells expressing the HcPT CDS was used for carrying out enzyme assays. Prenylation activity was tested using an array of xanthones, flavonoids, phenolic acids, and benzophenones in the presence of 0.2 mM dimethylallyl diphosphate (DMAPP). Among the examined substrates, 1,3,6,7-tetrahydroxyxanthone was preferred as prenyl acceptor (Supplementary Figure S2B). Product analysis demonstrated that the acceptor was prenylated once, which was detected by LC-MS in comparison with a sample of the authentic reference compound 1,3,6,7-tetrahydroxy-8-prenylxanthone (Supplementary Figure S3). The molecular ion peak [M + H]^+^ at *m*/*z* 329 and the MS/MS fragmentation pattern agreed with previous reports [16,35]. In HPLC-DAD, the enzymatic product co-eluted with the reference compound and the UV spectra matched, exhibiting the characteristic absorption bands of a tetraoxygenated xanthone skeleton at λ_max_ 254, 312, and 360 nm (Figure 4). Based on the R_t_ value, pyran ring formation was excluded (Figure 4C). Assays containing heat-denatured microsomes or the microsomal fraction from cells that harbored the empty vector served as control and lacked enzymatic product formation (Supplementary Figure S2B). For NMR analysis, the enzymatic product was purified from a large-scale incubation by semi-preparative HPLC on a C_18_ column (Experimental Section 3.10). The ^1^H-NMR data (Table 2) agreed with the previously published spectrum of 1,3,6,7-tetrahydroxy-8-prenylxanthone [35]. Alternative prenylation at the C–5 position was ruled out [36].

**Table 2:** ^1^H-NMR data of HcPT-formed 1,3,6,7-tetrahydroxy-8-prenylxanthone (600 MHz, acetone-*d*_6_/TMS).

Proton Position	δH *	*J* (Hz)	
1–OH	14.03 [1H]	s	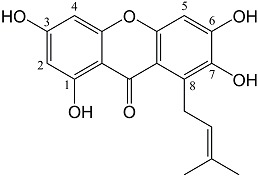
5–H	6.54 [1H]	s	
4–H	6.30 [1H]	d, 2	
2–H	6.15 [1H]	d, 2	
=CH–	5.47 [1H]	t, 6	
8–CH2	4.04 [2H]	d, 6	
=C(CH3)	1.80 [3H]	s	
=C(CH3)	1.63 [3H]	s	

* The signals of the aromatic protons were doubled (Δδ = 0.002–0.004 ppm), presumably because of the partial replacement of the hydroxyl group protons by deuterium due to the simultaneous presence of H_2_O and D_2_O in the solvent (isotope effects upon chemical shifts).

High C–8 prenylation activity was observed in 50 mM potassium phosphate buffer at pH 7.5 (Supplementary Figure S4A). No enzyme activity was detected in assays that lacked MgCl_2_, indicating the strict dependence of HcPT on the divalent cation Mg^2+^. Lower activities were observed with other divalent metal ions (Supplementary Figure S4B). The optimum incubation temperature was at 40 °C (Supplementary Figure S4C). The *K*_m_ values for 1,3,6,7-tetrahydroxyxanthone and DMAPP were 211 ± 16 and 87 ± 5 µM, respectively, as inferred from a Lineweaver-Burk plot. No appreciable loss in HcPT activity was observed upon storage at −80 °C for two months.

An array of prenylated xanthones, either naturally or synthetically generated, possess interesting pharmacological and biological activities, suggesting that a prenyl residue may significantly contribute [37,38,39]. An alkyl group at C–2 of 1,3-dihydroxy-2-methylxanthone was important for the selectivity against breast adenocarcinoma MCF-7 (Michigan Cancer Foundation-7) cells [40]. Furthermore, the anticholinesterase activity of 8-deoxygartanin was 17 times lower than that of γ-mangostin, whose IC_50_ value was 0.45 µg·mL^−1^, compared to 0.27 µg·mL^−1^ for galantamine [41]. The decrease in inhibition was attributed to the absence of the 8-prenyl moiety, as suggested by molecular docking. The enzymatic product of HcPT is a promising precursor for the partial synthesis of compounds that may offer an alternative for management of Alzheimer’s disease. Previous attempts to regiospecifically introduce prenyl moieties on the xanthone skeleton resulted in low yields. C–2 and C–4 prenylations of 1,3,5-trihydroxyxanthone were achieved at yields of 3% and 8%, respectively [42]. Furthermore, a modified method yielded 11% and 13%, respectively [43]. To date, there is no report about successful synthesis of a C–8 prenylxanthone. HcPT regiospecifically prenylated 1,3,6,7-tetrahydroxyxanthone at C–8 with 70% substrate conversion within two hours (Supplementary Figure S4D).

## 3. Experimental Section

### 3.1. Reagents

All reagents used were obtained from Roth (Karlsruhe, Germany), Sigma Aldrich (Steinheim, Germany), Acros Organics (Geel, Belgium), and Fisher Chemical (Nidderau, Germany), unless otherwise stated.

### 3.2. Synthesis of Trisammonium Dimethylallyl Diphosphate and 1,3,6,7-Tetrahydroxyxanthone

Trisammonium dimethylallyl diphosphate was synthesized as described previously [44], starting with commercially available dimethylallyl chloride. The substrate 1,3,6,7-tetrahydroxyxanthone was prepared semi-synthetically in a two-step synthesis. In a first step, 1,3,7-trihydroxyxanthone was synthesized according to Genoux-Bastide *et al.* [45] and purified over a silica gel column (silica gel 60, 0.040–0.063 mm (Merck, Darmstadt, Germany); column dimensions: 10 × 2.5 cm, 20 g silica gel) using gradient elution (20%–40% ethyl acetate in petroleum ether). The intermediate 1,3,7-trihydroxyxanthone was hydroxylated using a genetically modified yeast (*S. cerevisiae*) strain expressing a xanthone 6-hydroxylase gene. The resulting 1,3,6,7-tetrahydroxyxanthone was extracted from the growth medium and purified using semi-preparative thin layer chromatography (silica gel C_18_-100 UV_254_; methanol/water: 60:40).

### 3.3. Plant Material and Elicitation

Cell suspension cultures of *H. calycinum* L. (Hypericaceae) were generated as described previously [46]. For propagation, aliquots of 3 g of suction-dried cells were transferred every two weeks into 50 mL of liquid Linsmaier and Skoog (LS) medium [47]. Cultures were shaken in Erlenmeyer flasks at 125 rpm and 25 °C in the dark. Four-day-old cell cultures were treated with yeast extract as an elicitor (3 g·L^−1^) and harvested 8 h post-elicitation.

### 3.4. Isolation of an HcPT cDNA

Eight hours after yeast extract treatment, *H. calycinum* cell cultures were harvested by vacuum filtration and ground to fine powder under liquid nitrogen. Total RNA was extracted from 100 mg of homogenized plant material using the RNeasy Plant Mini Kit (Qiagen, Hilden, Germany). Oligo(dT)-primed reverse transcription was carried out at 42 °C with 1 µg total RNA and SuperScript II Reverse Transcriptase (Invitrogen, Karlsruhe, Germany). The synthesized cDNA pool served as template for amplifying the HcPT core fragment using the gene-specific primers 1 + 2 (Table 1, Supplementary Figure S1) and peqGOLD Taq-DNA-Polymerase (peqlab, Erlangen, Germany). Design of the primer pair was based on sequence information from a subtracted cDNA library [10]. After denaturation at 94 °C (2 min), 30 cycles were carried out at 94 °C (30 s), 55 °C (45 s), and 72 °C (2 min), followed by final extension at 72 °C for 10 min. The core fragment was then extended toward the 5′ and 3′ ends by rapid amplification of cDNA ends (RACE), using the protocol of the SMARTer RACE cDNA Amplification Kit (Clontech, Heidelberg, Germany) and the gene-specific primers 3 + 4 (Table 1, Supplementary Figure S1). The resulting amplicons were cloned into the pGEM-T Easy vector (Promega, Mannheim, Germany) and sequence information was obtained by DNA sequencing at GATC-Biotech (Konstanz, Germany).

### 3.5. Semi-Quantitative Reverse Transcription PCR

At defined times (0–20 h) after yeast treatment, total RNA was isolated from cultured *H. calycinum* cells (0.5 mg). Reverse transcription and PCR were carried out as described above under section 3.4 except that the annealing time and amplification cycles were reduced to 30 s and 28 cycles, respectively. Primer pair 1 + 2 (Table 1, Supplementary Figure S1) was used to analyze HcPT transcripts. To examine the HcCNL transcript level, primers 5 + 6 were used. The 18S ribosomal DNA (*Hypericum* rDNA; AF206934) transcripts served to normalize RT-PCR results using primer pair 7 + 8. Annealing temperatures for HcPT, HcCNL and 18S were 55, 60 and 51 °C, respectively. The PCR products were finally analyzed as described under 3.4 using the free open source image processing program ImageJ (version 1.36, http://rsb.info.nih.gov/ij/) [10].

### 3.6. Heterologous Expression of HcPT

Within the first expression trials, prokaryotic cells from the LEMO21 (DE3) system (New England Biolabs, Frankfurt, Germany) were used according to the manufacturer’s protocol using the pRSET-B expression vector and the overexpression primers 9 + 10 (Table 1, Supplementary Figure S1). HcPT expression in the yeast strain INVSc1 (*S. cerevisiae*, Invitrogen, Karlsruhe, Germany) was carried out as described elsewhere [48]. The expression plasmid pESC-URA and a primer pair 11 + 12 with an *Eco*RI and a *Pac*I restriction site were used for cloning and expression analysis. For overexpression in insect cells (*S. frugiperda*, *Sf9)*, the HcPT CDS was amplified with Phusion Hot Start II High-Fidelity DNA Polymerase (Thermo Scientific, Dreieich, Germany) using the primers 13 + 14 with an *Eco*RI restriction site and a *Hin*dIII restriction site, respectively. PCR included initial denaturation at 98 °C (30 s), 35 cycles with 98 °C (10 s), 66.1 °C (30 s), and 72 °C (60 s), followed by final extension at 72 °C (10 min). Subcloning into the pFastBac1 expression vector (Invitrogen, Karlsruhe, Germany) was made by ligating the *Eco*RI-*Hin*dIII-digested PCR product into *Eco*RI-*Hin*dIII-linearized pFastBac1, generating the expression plasmid pFastBac1-HcPT. The nucleotide sequence of the expression construct was verified by DNA sequencing. HcPT-baculovirus was generated according to the Bac-to-Bac Baculovirus Expression System manual (Invitrogen, Karlsruhe, Germany). For cultivation of *Sf9* insect cells, *Sf-*900 II serum-free medium was supplemented with 1% penicillin/streptomycin and 10% fetal calf serum. Insect cell cultures were shaken at 27 °C at 140 rpm and were diluted to a concentration of 2 × 10^6^ cells mL^−1^ for infection. Cell suspension volumes between 20 and 200 mL were infected with an HcPT-baculovirus stock.

### 3.7. Microsomal Preparation from Insect Cells

Microsomes were isolated as described previously [22], except for minor modifications. The infected cell suspension was transferred into 50 mL tubes and was pelleted at 2500× *g* for 5 min at 4 °C. The pellet was re-suspended in 5 mL of sonication buffer. The resulting cell suspension was sonicated for 60 s three times (duty cycle: 15%, output control: 1.5) using a Sonifier 250 (Branson, Danbury, CT, USA). After centrifugation under the above conditions for removing cell debris and nuclei, the microsomal pellet was obtained by ultracentrifugation of the supernatant at 100,000× *g* for 1 h at 4 °C. The pellet was re-suspended in 2 mL of ice-cold buffer A by pipetting with syringe and needle to form the enzyme solution for the PT assay. Protein concentrations were determined by the Bradford method [49] with bovine serum albumin as standard.

### 3.8. In Vitro Enzyme Assay

The standard PT incubation contained 0.2 mM 1,3,6,7-tetrahydroxyxanthone, 0.2 mM dimethylallyl diphosphate, 4 mM MgCl_2_, 0.5 mM dithiothreitol, and an aliquot of the microsomal fraction containing around 100 µg protein. The final assay volume was adjusted to 250 µL with 50 mM potassium phosphate buffer (pH 7.5). After incubation at 40 °C for 30 min, the reaction was stopped by the addition of 25 µL glacial acetic acid (20% in methanol). The reaction mixture was extracted twice with ethyl acetate (500 and 250 µL) and the combined organic phase was evaporated to dryness under an air stream at room temperature. The residue was dissolved in 80 µL methanol (HPLC grade) and analyzed by HPLC. To determine the *K*_m_ value for the substrate 1,3,6,7-tetrahydroxyxanthone, concentrations from 5 to 1000 µM were used, while keeping the DMAPP concentration constant at 400 µM. To measure the *K*_m_ value for the prenyl donor DMAPP, the concentration varied from 5–500 µM, with the 1,3,6,7-tetrahydroxyxanthone level being constant at 500 µM.

### 3.9. HPLC and LC-ESI-MS Analyses

The reaction product was analyzed by HPLC (Agilent 1260 Infinity Quaternary LC System; Agilent Technologies, Santa Clara, CA, USA) equipped with a diode array detector (G4212A). A ZORBAX SB-C_18_ column (3.5 µm, 4.6 × 150 mm; Agilent) was used. The mobile phase consisted of water acidified with 1 mM formic acid and methanol. For standard analysis at a flow rate of 0.5 mL·min^−1^, the methanol concentration changed as follows: 50%–75% in 5 min, 75%–90% in 10 min, and 95% in 5 min. To analyze incubations containing 1,7-dihydroxyxanthone as a substrate, methanol was replaced with acetonitrile in the following gradient: 40%–70% in 5 min, 70%–92% in 5 min, and 92%–95% in 15 min. The detection wavelength used for monitoring the elution of both 1,3,6,7-tetrahydroxyxanthone and its prenylated product was 254 nm. To analyze the potential conversion of other substrates, the incubation time was elongated to 2 h and corresponding absorption maxima were chosen as detection wavelengths. 1,3,6,7-Tetrahydroxy-8-prenylxanthone was identified by ESI-MS analysis, as described previously [10]. The molecular ion peak [M + H]^+^ of the enzymatic product was further analyzed by MS/MS experiments in the enhanced product ion mode of the instrument using nitrogen gas for collision-induced dissociation at the high-level setting. The Analyst software (version 1.4.2; Applied Biosystems/MDS Sciex, Waltham, MA, USA) served for data acquisition and processing (Supplementary Figure S3).

### 3.10. ^1^H-NMR Analysis

To achieve sufficient amounts of 1,3,6,7-tetrahydroxy-8-prenylxanthone, the volume of the standard enzyme assay was increased 200-fold and the incubation time at 40 °C was extended to 20 h, adding after 3 h another 25% protein and DMAPP relative to the starting amounts. ^1^H-NMR analysis was carried out on a Bruker Avance II 600 spectrometer using acetone-*d*_6_ (Deutero, Kastellaun, Germany) as solvent and 20 °C as sample temperature.

## 4. Conclusions

An aromatic prenyltransferase (HcPT) specific for enzymatic alkylation of xanthones has been isolated from elicitor-treated *H. calycinum* cell cultures. The enzyme regiospecifically introduced a prenyl side chain at C–8 of the dibenzo-γ-pyrone skeleton. Recently, classification and chemical synthesis of simple and prenylated xanthones have been reviewed [50,51,52,53] and divergent biological activities have been reported [54,55,56]. The HcPT product 1,3,6,7-tetrahydroxy-8-prenylxanthone shares structural features with γ-mangostin, a potent inhibitor of cholinesterase. The importance of the 8-prenyl group was revealed by docking studies [41]. Thus, HcPT offers an interesting tool to provide a precursor for semisynthesis of pharmacologically active compounds with the potential to be used in the treatment of Alzheimer’s disease. In addition, further xanthone-specific prenyltransferases need to be studied at the gene level to pave the way for biotechnological production of a wider array of compounds, which may improve the therapy of neurodegenerative disorders.

Sequence data from this article can be found in the GenBank/EMBL data libraries under the accession number KT325851.

## Supplementary Materials

Supplementary materials can be accessed at: http://www.mdpi.com/1420-3049/20/09/15616/s1.
