# Supplementary material for: Molecular Cloning and Characterization of a Xanthone Prenyltransferase from Hypericum calycinum Cell Cultures
Source: Molecules. 2015 Aug 27;20(9):15616–30. doi: 10.3390/molecules200915616 (PMC6332024; doi:10.3390/molecules200915616)
Supplement: Supplementary file 1 [file molecules-20-15616-s001.pdf]

Supplementary Materials

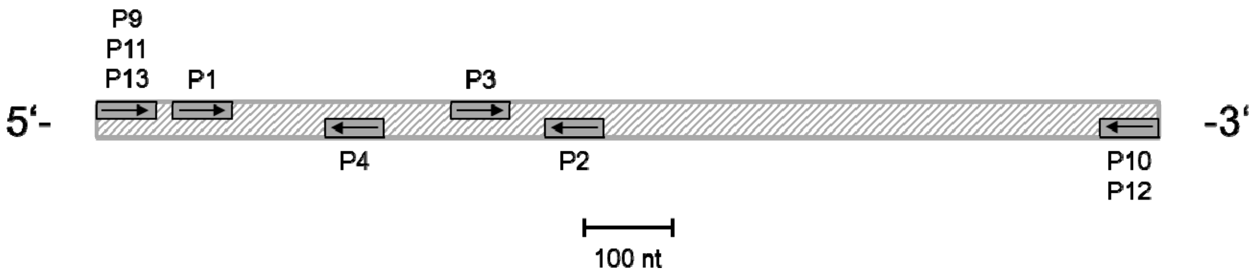

Figure S1. HcPT-specific primer-binding positions. Dark boxes highlight the specific binding sites, arrowheads indicate the 5′→3′ orientation of the oligonucleotides. →: forward primer, ←: reverse primer, nt: nucleotides.

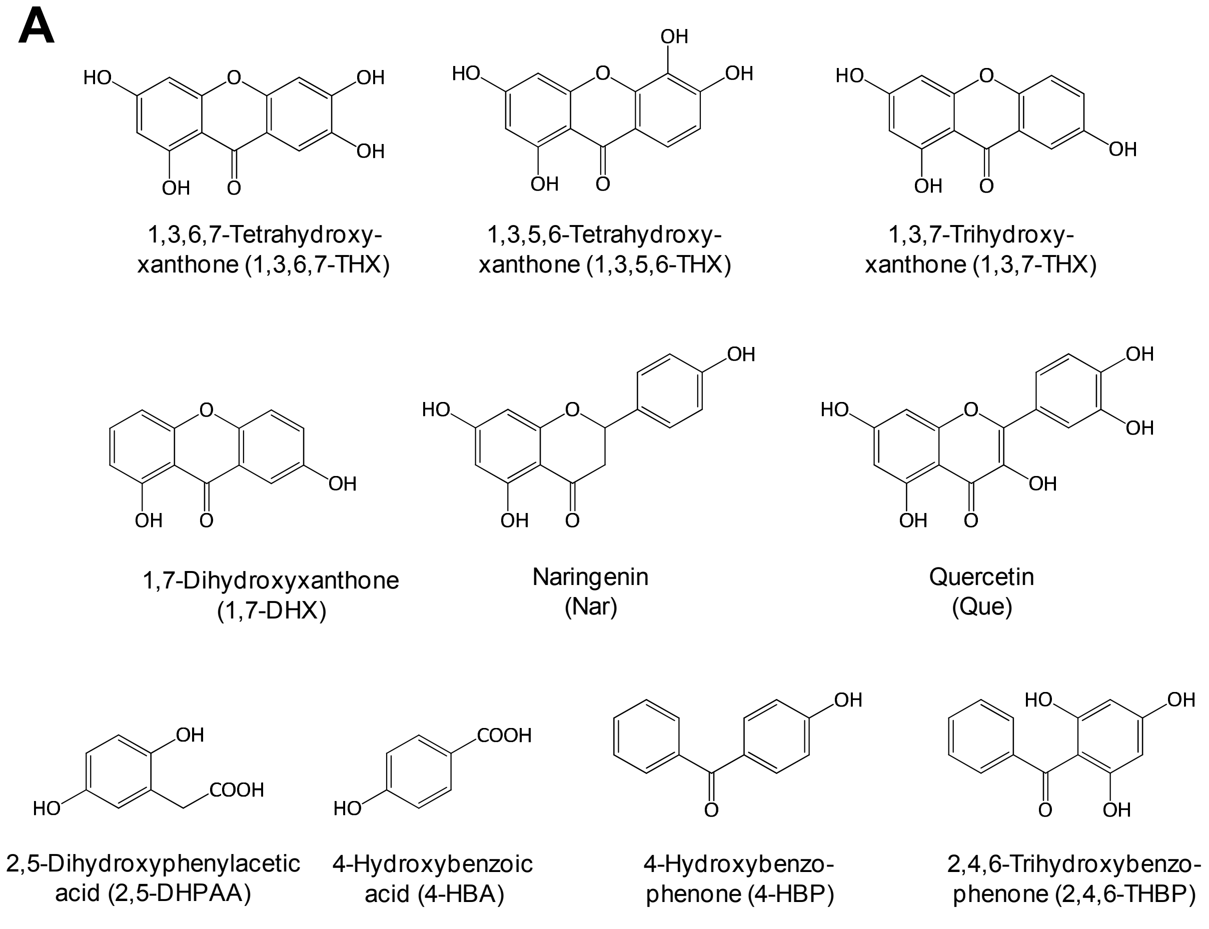

Figure S2. *Cont.*

B

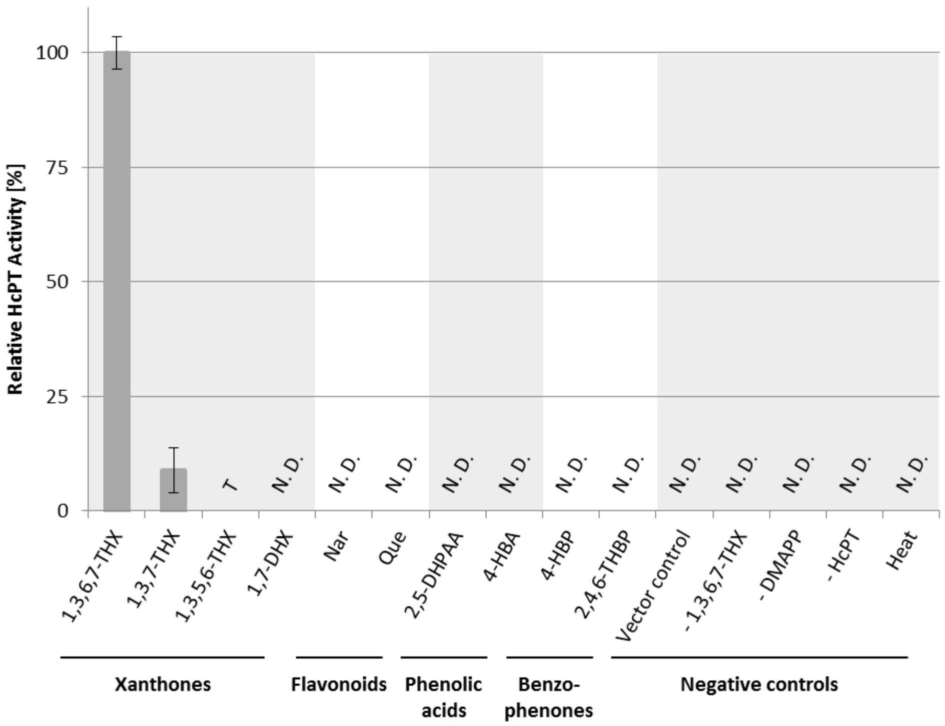

Figure S2. Substrate specificity of recombinant HcPT. (**A**) Chemical structures of aromatic substrates tested; (**B**) Dimethylallyl transferase activity of HcPT with various prenyl acceptors. Prenylation of 1,3,6,7-THX is set as 100%. T: trace activity, N.D.: not detected, 1,3,6,7-THX: 1,3,6,7-tetrahydroxyxanthone, 1,3,7-THX: 1,3,7-trihydroxyxanthone, 1,3,5,6-THX: 1,3,5,6-tetrahydroxyxanthone, 1,7-DHX: 1,7-dihydroxyxanthone, Nar: naringenin, Que: quercetin, 2,5-DHPAA: 2,5-dihydroxyphenylacetic acid, 4-HBA: 4-hydroxybenzoic acid, 4-HBP: 4-hydroxybenzophenone, 2,4,6-THBP: 2,4,6-trihydroxybenzophenone, Vector control: standard assay with a preparation using an empty expression vector, -1,3,6,7-THX: standard assay without prenyl acceptor, -DMAPP: standard assay without dimethylallyl diphosphate, -HcPT: standard assay without HcPT, Heat: standard assay with denatured enzyme (95 °C for 30 min under vigorous vortexing).

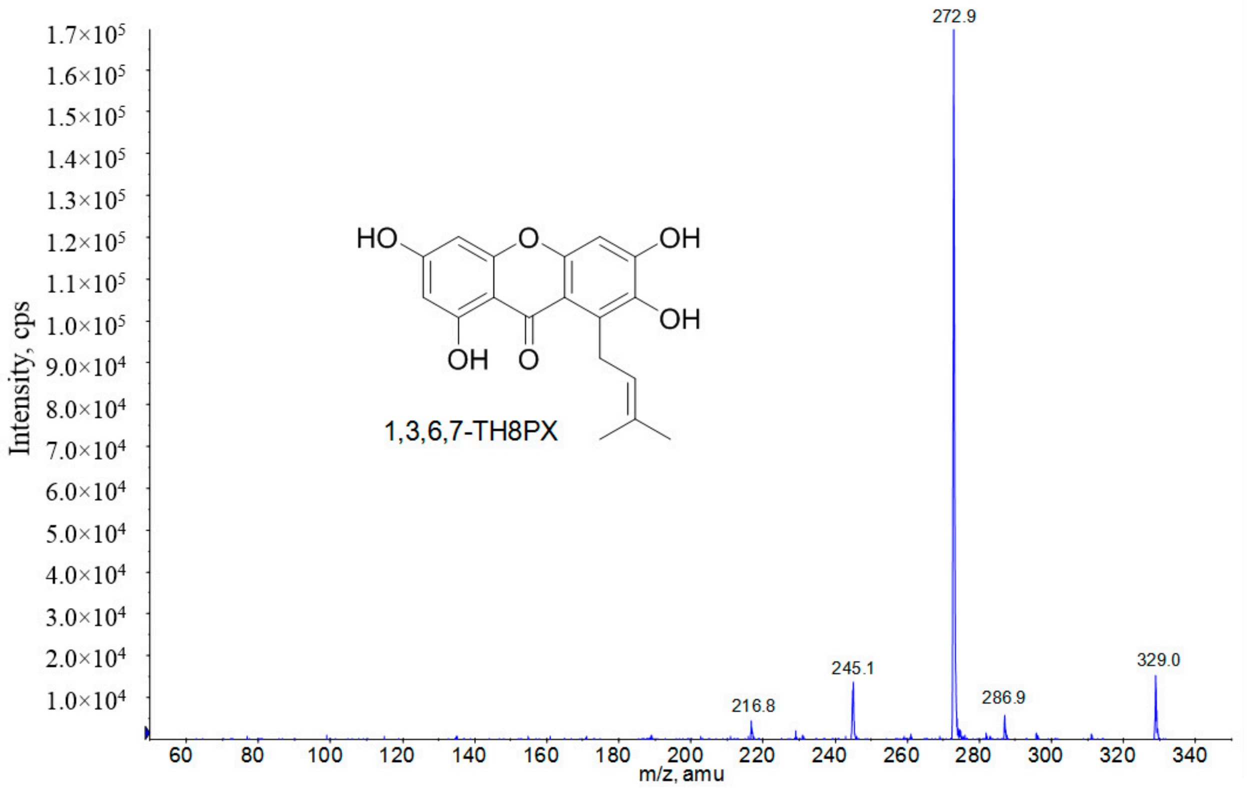

Figure S3. Fragmentation mass spectrum of the HcPT-formed protonated molecular ion of 1,3,6,7-tetrahydroxy-8-prenylxanthone ($[M + H]^+ = 329$) in positive-ion electrospray ionization enhanced product ion mass spectrometry (ESI-EPI-MS). 1,3,6,7-TH8PX: 1,3,6,7-tetrahydroxy-8-prenylxanthone.

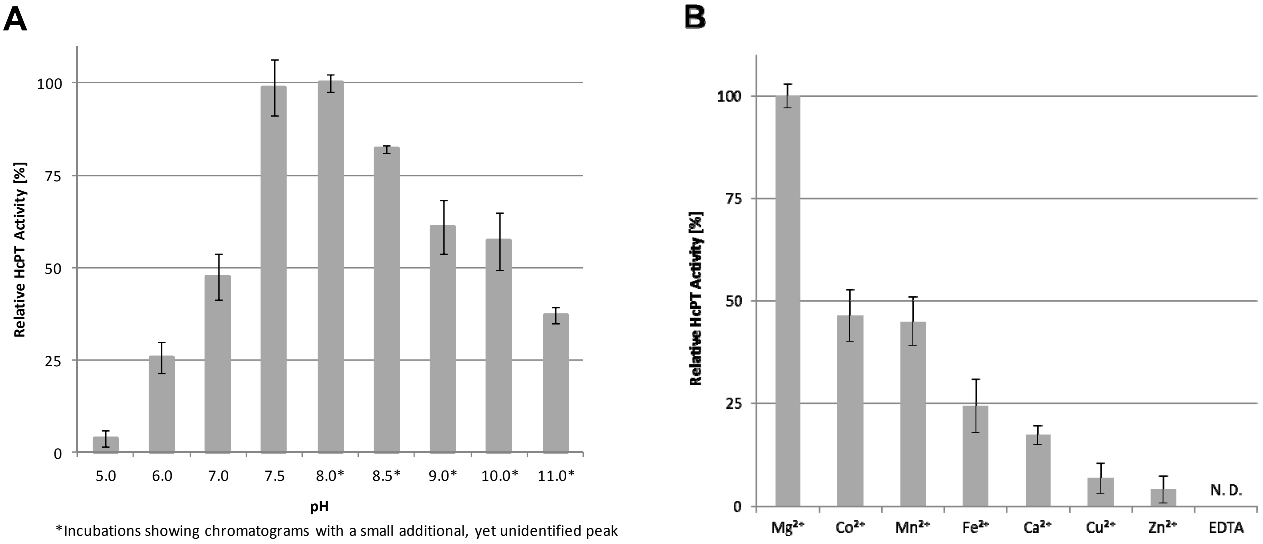

Figure S4. *Cont.*

C

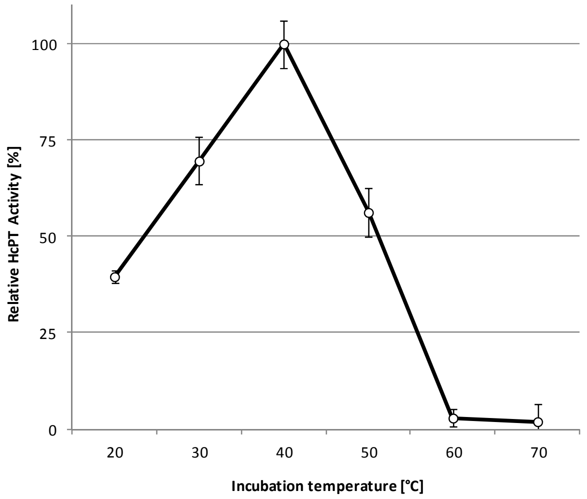

D

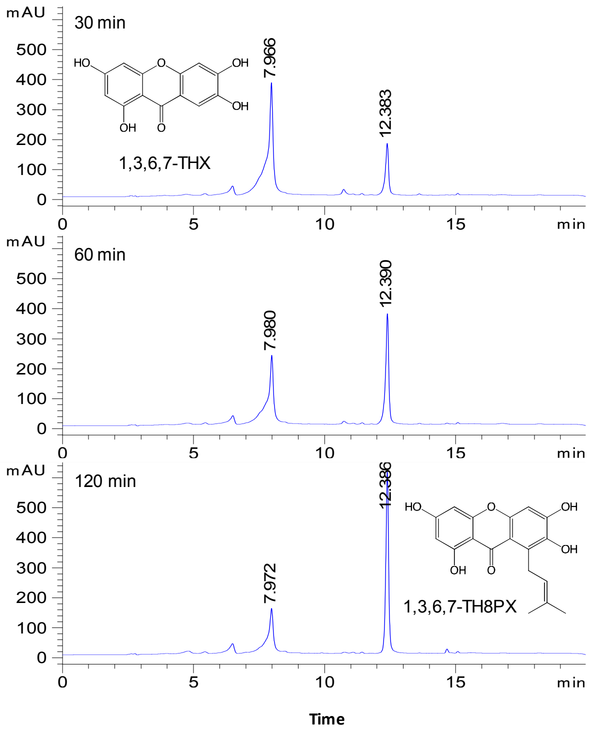

Figure S4. HPLC analysis of HcPT activity dependent on the incubation conditions. (**A**) pH dependency determined in standard incubations with varying pH values. Highest product formation was set as 100%; (**B**) Requirement for divalent cations. HcPT activity was tested in standard incubations either containing various metal ions (Mg^{2+}, Co^{2+}, Mn^{2+}, Fe^{2+}, Ca^{2+}, Cu^{2+}, Zn^{2+}) or lacking divalent cations (replaced by EDTA). Activity in the presence of Mg^{2+} was set as 100%; (**C**) Temperature-related formation of prenylated 1,3,6,7-THX in standard assays, with maximum activity at 40 °C; (**D**) HPLC chromatograms indicating the time-dependent prenylation of 1,3,6,7-tetrahydroxyxanthone by changes in the substrate peak-product peak-ratio (standard incubations). Detection wavelength: 254 nm. N.D.: Not detected. 1,3,6,7-THX: 1,3,6,7-tetrahydroxyxanthone, 1,3,6,7-TH8PX: 1,3,6,7-tetrahydroxy-8-prenylxanthone.
